# Cytotoxicity of 5-(3-methyl-1-triazeno)imidazole-4-carboxamide (MTIC) on Mer+, Mer+Rem- and Mer- cell lines: differential potentiation by 3-acetamidobenzamide.

**DOI:** 10.1038/bjc.1988.8

**Published:** 1988-01

**Authors:** J. M. Lunn, A. L. Harris

**Affiliations:** Cancer Research Unit, University of Newcastle upon Tyne, Royal Victoria Infirmary, UK.

## Abstract

Mechanisms of resistance to the active metabolite 5-(3-methyl-1-triazeno)imidazole-4-carboxamide (MTIC) of the drug 5-(3,3-dimethyl-1-triázeno)imidazole-4-carboxamide (DTIC) were studied in three human cell lines with differing amounts of the repair enzyme O6-alkylguanine-DNA alkyltransferase (O6AT). The lines were HT29 (Mer+Rem+), A549 (Mer+Rem-) and VA13 (Mer-). The ability to repair O6 methyl-guanine was directly related to resistance to MTIC (HT29 ID50 650 mumol l-1, A549 ID50 210 mumol l-1, VA13 ID50 15 mumol l-1. MTIC produced DNA single strand breaks over the range of one log of cell kill, but depletion of cellular NAD levels could not be detected until there was greater than 95% cell kill. Inhibitors of the repair enzyme adenosine diphosphoribosyl transferase (ADPRT) potentiated killing by 2-fold in the Mer+ cell lines but not the Mer- line. The enhancement was directly proportional to an increase in DNA strand breaks but not a change in their half-life. Therefore resistance to the clinically used methylating agent MTIC can be partly overcome by inhibiting ADPRT but a role for ADPRT as a suicide mechanism in response to alkylating agent damage is unlikely.


*Br. J. Cancer* (1988), **57**, 54–58
© The Macmillan Press Ltd., 1988

### Cytotoxicity of 5-(3-methyl-1-triazeno)imidazole-4-carboxamide (MTIC) on Mer$^+$, Mer$^+$Rem$^-$ and Mer$^-$ cell lines: Differential potentiation by 3-acetamidobenzamide

J.M. Lunn & A.L. Harris

*Cancer Research Unit, University of Newcastle upon Tyne, Royal Victoria Infirmary, Newcastle upon Tyne NE1 4LP, UK.*

**Summary** Mechanisms of resistance to the active metabolite 5-(3-methyl-1-triazeno)imidazole-4-carboxamide (MTIC) of the drug 5-(3,3-dimethyl-1-triazeno)imidazole-4-carboxamide (DTIC) were studied in three human cell lines with differing amounts of the repair enzyme O$^6$-alkylguanine-DNA alkyltransferase (O$^6$AT). The lines were HT29 (Mer$^+$Rem$^+$), A549 (Mer$^+$Rem$^-$) and VA13 (Mer$^-$). The ability to repair O$^6$ methylguanine was directly related to resistance to MTIC (HT29 ID50 650$\mu$mol l$^{-1}$, A549 ID50 210$\mu$mol l$^{-1}$, VA13 ID50 15$\mu$mol l$^{-1}$). MTIC produced DNA single strand breaks over the range of one log of cell kill, but depletion of cellular NAD levels could not be detected until there was greater than 95% cell kill. Inhibitors of the repair enzyme adenosine diphosphoribosyl transferase (ADPRT) potentiated killing by 2-fold in the Mer$^+$ cell lines but not the Mer$^-$ line. The enhancement was directly proportional to an increase in DNA strand breaks but not a change in their half-life. Therefore resistance to the clinically used methylating agent MTIC can be partly overcome by inhibiting ADPRT but a role for ADPRT as a suicide mechanism in response to alkylating agent damage is unlikely.

One of the few active drugs in the treatment of malignant melanoma (Comis, 1976) and a component of sarcoma treatment regimens (Gottlieb *et al.*, 1976) is the substituted triazene 5-(3,3-dimethyl-1-triazeno)imidazole-4-carboxamide (DTIC). The mechanism of action is thought to involve metabolic N-demethylation to give the cytotoxic monomethyl triazene 5-(3-methyl-1-triazeno)imizadole-4-carboxamide (MTIC), which can methylate N-7 sites of guanine in DNA (Preussman & von Hodenburg, 1970).

Because *de novo* or secondary resistance to DTIC is a common problem, we have investigated the mechanisms of resistance to killing and ways to potentiate cytotoxicity in human tumour cell lines.

Alkylation of DNA at the O$^6$ position of guanine may be a particularly important lesion in determining cellular sensitivity to methylating agents (Erickson *et al.*, 1980). Human tumour cells which possess the suicide repair enzyme O$^6$-alkylguanine-DNA alkyltransferase (O$^6$AT) can remove O$^6$ alkyl lesions by an error-free repair mechanism and are much more resistant to methylating agents than cells deficient in this enzyme (Harris *et al.*, 1983). Cell lines capable of supporting the growth of N-methyl-N′-nitro-N-nitrosoguanidine (MNNG) damaged adenovirus are also resistant to MNNG cytotoxicity, possess O$^6$AT and are defined as having the Mer$^+$ phenotype (Scudiero *et al.*, 1984). Mer$^-$ tumour strains are unable to repair O$^6$ methylguanine (O$^6$MeG) lesions in DNA. An intermediate group of Mer$^+$ cell lines, although able to support the growth of MNNG-treated adenovirus, have an intermediate sensitivity to the cytotoxicity of MNNG and are designated Rem$^-$ (Scudiero *et al.*, 1984).

The Mer phenotypes also correlate with sensitivity to crosslinking nitrosoureas such as 1,3-bis(2-chloroethyl)-1-nitrosourea (BCNU) and 1-(2-chloroethyl)-1-nitrosourea (CNU) and the methylating agent methyl methanesulphonate (MMS). DNA crosslinking by chloroethylnitrosoureas is believed to involve the initial formation of a monoadduct at the O$^6$-position of guanine residues. Removal of this monoadduct by O$^6$AT prevents crosslink formation (Erickson *et al.*, 1980).

MTIC has recently been shown to alkylate DNA in the guanine–O$^6$ position *in vivo* (Meer *et al.*, 1986), so we have compared the sensitivity of cell lines with the three different Mer phenotypes.

Correspondence: J.M. Lunn.
Received 17 March 1987; and in revised form, 11 August 1987.

Methylating agents also interact with another component of DNA repair – poly ADP-ribose polymerase, or adenosine diphosphoribosyl transferase (ADPRT) (Durkacz *et al.*, 1980). This enzyme is absolutely dependent on DNA strand breaks for activity and catalyses the conversion of nicotinamide adenine dinucleotide (NAD) to poly ADP-ribose, which covalently modifies numerous nuclear proteins (Creissen & Shall, 1982). Inhibition of ADPRT activity with nicotinamide analogues, such as 3-aminobenzamide (3AB), potentiates the killing effects of several methylating agents, and it has been postulated that this is due to the regulation of DNA ligases by ADPRT. Strand breaks are induced by the repair and spontaneous loss of methylated bases in DNA, and these breaks accumulate in the presence of ADPRT inhibitors. The depletion of NAD in synthesizing poly ADP-ribose has been postulated to lead to a drop in ATP levels and hence cell death (Sims *et al.*, 1983).

A more potent nicotinamide analogue is 3-acetamidobenzamide (3AAB) (Purnell & Whish, 1980). We have therefore compared the potentiating effects of 3AAB on cell lines with differing sensitivities to MTIC to try and overcome resistance and related the effects to strand breaks and NAD levels.

### Materials and methods

### *Cell culture*

HT29 (human colon carcinoma) cells were were obtained from the American Type Culture Collection (ATCC HTB-38). A549 (human lung adenocarcinoma) cells were kindly supplied by Dr Adi Gazdar, NCI, Bethesda, Maryland, USA. WI38-VA13 (SV40-transformed WI38 human fibroblast) cells were obtained from Flow Laboratories Ltd., Rickmansworth, Herts, WD3 1PQ.

Cells were grown as monolayers in either Roswell Park Memorial Institute Medium 1640 (A549 cells) or Eagle's Minimum Essential Medium (HT29 and VA13 cells). Both media were supplemented with 10% (v/v) foetal bovine serum, penicillin (100 IU ml$^{-1}$), streptomycin (100$\mu$g ml$^{-1}$) and nystatin (50 IU ml$^{-1}$). EMEM was further supplemented with non-essential amino acids (0.1 mmol l$^{-1}$).

### *Drugs*

MTIC was synthesized by preparing the diazo derivative (DZC) of 5-aminoimidazole-4-carboxamide (AICA) and reacting this with methylamine in dimethyl sulphoxide (DMSO). Product purity was established by NMR spectroscopy. A full description of the synthesis will be published separately (Bleasdale *et al.*, in preparation). MTIC was stored protected from light at −20°C. Because of its short half-life in aqueous solutions, MTIC was dissolved in DMSO immediately before use at 200 times the desired final concentrations and added to cell cultures.

3AAB was synthesized by reacting 3AB with acetic anhydride (Purnell & Whish, 1980).

*Cell survival assay*

Assays were carried out in triplicate. Cells ($\sim 30 \times 10^3$) were seeded into 6-well culture dishes 1–2 days before use. Culture medium was replaced just before the addition of solutions of MTIC in DMSO. After 30 min at 37°C, medium containing or omitting 3AAB was replaced and cells were grown for about three doublings. Cells were harvested by trypsinization and counted using a Coulter ZM counter. Cell survival was assessed by the increase in number of cells, expressed as a percentage of the mean value for cultures not receiving MTIC.

*DNA strand break assay*

An alkaline unwinding procedure was used to measure DNA strand breaks (Cavanaugh *et al.*, 1984). Cells were grown in 100 x 15 mm tissue culture dishes to give $\sim 5 \times 10^6$ cells/dish. At appropriate times after addition of MTIC, growth medium was aspirated, the cell layer was rinsed with ice-cold PBS and the cells were harvested using a cell scraper (Costar). The number of DNA strand breaks was calculated per alkaline unwinding unit (Cavanaugh *et al.*, 1984).

*NAD assay*

Cellular NAD levels were measured using the procedure of Nisselbaum and Green(1969). Adherent cell cultures were exposed to MTIC, and NAD levels were measured after 2 h (Skidmore *et al.*, 1979). No change of growth medium was necessary during this time, since no active drug remained after 15 min in culture medium (Parsons *et al.*, 1982, and our own unpublished findings). Cell layers were rinsed with ice-cold PBS and harvested in 50% aqueous ethanol using a cell scraper (Costar). Cell suspensions were disrupted by sonication (MSE Soniprep 150) and centrifuged (MSE Microfuge). The supernatant solutions were assayed for NAD.

### Results

*MTIC cytotoxicity in $Mer^+$, $Mer^+Rem^-$, $Mer^-$ cell lines*

Cell survival was measured in HT29 ($Mer^+Rem^+$), A549 ($Mer^+Rem^-$) and VA13 ($Mer^-$) cell lines. The results from representative experiments are shown in Figures 1, 2 and 3. The sensitivity was inversely proportional to the ability to remove $O^6$ methylguanine, with doses producing 50% toxicity (ID50) of $650 \pm 140\,\mu mol\,l^{-1}$, $210 \pm 40\,\mu mol\,l^{-1}$ and $15 \pm 4\,\mu mol\,l^{-1}$ for HT29, A549 and VA13 cells respectively. Since MTIC is rapidly hydrolysed to AICA, which, when ribosylated (AICAR), could have an effect on purine precursor pools, the effect of AICAR was assessed and found to be non-cytotoxic at equimolar concentrations to MTIC (Figure 2).

*Potentiation of MTIC by 3AAB*

3AAB potentiated cell killing by a dose enhancement factor of 2 at 50% survival in both $Mer^+$ cell lines. At MTIC doses producing 1% survival in the presence of 3AAB in both cell lines, there was approximately 30% survival in the absence of 3AAB.

In contrast, there was no potentiating effect of 3AAB on the $Mer^-$ cell line until less than 50% survival was obtained.

At low survival, there was a potentiating effect consisting of a 4-fold difference in survival at $50\,\mu mol\,l^{-1}$ MTIC (3% with 3AAB, 11% without 3AAB).

**Figure 1** Effect of 3AAB on the cytotoxicity of MTIC:HT29 cells. The effect on cell proliferation of each concentration of drug was measured in triplicate cultures. Mean values ± s.d. are shown. ● MTIC alone, ○ MTIC + 1 mmol l⁻¹ 3AAB.

**Figure 2** Effect of 3AAB on the cytotoxicity of MTIC:A549 cells. The effect on cell proliferation of each concentration of drug was measured in triplicate cultures. Mean values ± s.d. are shown. ● MTIC alone, ○ MTIC + 1 mmol l⁻¹ 3AAB, △ AICAR.

*Relation of DNA strand breaks to cell killing and potentiation by 3AAB*

DNA strand breaks were assayed over the range of MTIC concentrations associated with up to approximately one lethal hit per cell (D37) in the $Mer^+$ cell line A549. This range is likely to be relevant to *in vivo* use of the drug where only a 1–2 log cell kill can be achieved in responding tumours.

The assay was linear up to $500\,\mu mol\,l^{-1}$ MTIC, but extensive fragmentation beyond $600\,\mu mol\,l^{-1}$ prevented quantitation of strand breaks at high MTIC concentrations

**Figure 3** Effect of 3AAB on the cytotoxicity of MTIC:VA13 cells. The effect on cell proliferation of each concentration of drug was measured in triplicate cultures. Mean values ± s.d. are shown. ● MTIC alone, ○ MTIC + 1 mmol l$^{-1}$ 3AAB.

(Figure 4). Thus, over the range of concentrations likely to be relevant *in vivo*, there was a linear relationship between strand breaks and MTIC concentration.

It has been hypothesized that inhibitors of ADPRT enhance the action of cytotoxic drugs by interfering with DNA repair (Shall, 1982). Therefore, DNA strand breaks were measured in A549 cells exposed to 100 μmol l$^{-1}$ MTIC in the absence or presence of 3AAB (Figure 5). The time course of appearance of strand breaks in the absence of 3AAB was rapid and they disappeared in a biphasic manner, with an initial half-life of 50 min.

In the presence of 3AAB there was a doubling of the peak number of strand breaks detected, but their rate of disappearance was not decreased. The initial half-life of disappearance was 45 min.

Thus, the dose enhancement factor was proportional to the increase in peak number of strand breaks, not a change in half-life.

**Figure 4** DNA strand breaks produced in A549 cells by MTIC. DNA strand breaks produced by 30 min exposure to a range of concentrations of MTIC were assessed in triplicate cultures. Mean values are shown.

**Figure 5** DNA strand breaks produced in A549 cells by MTIC. Appearance and removal of DNA strand breaks following exposure of cells to MTIC (100 μmol l$^{-1}$) in the absence (●) or presence (○) of 3AAB (1 mmol l$^{-1}$).

### *Effects of MTIC on NAD levels*

Since the activation of ADPRT, caused by the appearance of DNA strand breaks, would result in the utilization of NAD for the synthesis of poly ADP-ribose, the dose-related effects of MTIC on NAD levels were assessed. MTIC produced a marked lowering in NAD levels in A549 cells linearly related to dose (Figure 6). However, this decrease was over the concentration range 1–5 mmol l$^{-1}$ and did not occur below 1 mmol l$^{-1}$ MTIC, a concentration associated with >95% cell killing.

**Figure 6** Effect of MTIC on NAD levels in A549 cells. NAD was measured in triplicate cultures 2 h after exposure to a range of concentrations of MTIC. Mean values ± s.d. are shown.

### Discussion

MTIC produced differential effects on cell lines with the different Mer phenotypes. The proportional sensitivity of the three lines to MTIC ($Mer^+$ 1, $Mer^+Rem^-$ 0.3, $Mer^-$ 0.02) is very similar to that reported for MNNG ($Mer^+$ 1, $Mer^+Rem^-$ 0.26, $Mer^-$ 0.03) (Scudiero *et al.*, 1984). It is apparent that variations in the $Mer^+$ phenotype ($Rem^-$ or $Rem^+$) are associated with differences in sensitivity to MTIC. This is potentially important clinically, because the $Mer^-$ phenotype has not been convincingly demonstrated in primary human tumours. Although approximately 30% of cell lines studied are $Mer^-$, in studies of human normal and tumour material directly, absence of $O^6$AT is much rarer (Waldstein *et al.*, 1982; Myrnes *et al.*, 1984*a*, *b*; Gerson *et al.*, 1985; Wiestler *et al.*, 1984; Umbenhauer *et al.*, 1985; Grafstrom *et al.*, 1984; Wani *et al.*, 1985). In none of the series is absence reported, out of over 150 patient samples. Also, SV40 transformation of $Mer^+$ cell lines can produce the $Mer^-$ phenotype (Day *et al.*, 1980).

Therefore attempts to potentiate cell killing in $Mer^+$ cell lines are much more important for therapeutic applications. Potentiation of MTIC toxicity by 3AAB was similar with cells of both $Mer^+$ phenotypes. Since $O^6$MeG is not removed from DNA by excision repair (Olsson & Lindahl, 1980), this suggests that it is the production of AP sites, and hence DNA strand breaks, during excision repair of other bases that is important in killing $Mer^+$ cell lines and that the level of these breaks is increased in the presence of 3AAB. These experiments were all carried out in a concentration range of drugs relevant to the proportional cell kill obtained *in vivo* with chemotherapy. In this cytotoxic drug range there were two observations at conflict with some current assessments of the interaction of ADPRT with inhibitors and DNA repair.

Although 3AAB doubled the amount of strand breaks at $100\,\mu mol\,l^{-1}$ MTIC and produced a 2-fold dose enhancement effect, there was no change in the rate of repair of breaks. Since one major explanation of the effect of 3AAB is that DNA ligase II is not activated for DNA repair when ADPRT is inhibited (Creissen & Shall, 1982), one would expect a decreased rate of repair. Recently, Walker *et al.* (1984) and Cleaver and Morgan (1985) showed that more repair patches result from DNA damage in the presence of 3AB, rather than longer patches that would occur if ligation was decreased. Similarly, Moran and Ebisuzaki (1985) found that 3AB produced an increase in strand breaks but no change in rejoining rate. Our results with MTIC are compatible with the postulate that inhibitors of ADPRT allow the action of an endonuclease on damaged DNA, producing more breaks but not changing patch size of rate of ligation.

The other proposed mechanism relating increased cell killing to the use of ADPRT inhibitors involves prevention of NAD depletion. NAD depletion does occur in methylating agent treated cells, and can ultimately lead to a decrease in intracellular ATP levels. It has been postulated that this is a suicide mechanism for badly damaged cells and that ATP depletion may allow repair to occur, but not replication (Wintersberger & Wintersberger, 1985; Carson *et al.*, 1986; Sims *et al.*, 1985). Stopping the NAD drop by inhibiting ADPRT would maintain ATP levels and allow replication on a damaged template, hence potentiating the effects of DNA damage.

Although we showed that MTIC, similarly to other methylating agents, did produce a NAD drop, this only occurred at very high MTIC levels ($>1\,mmol\,l^{-1}$). Thus the postulated mechanism of action of ADPRT inhibitors is unlikely to be relevant *in vivo*.

Parsons *et al.* (1982) and Hayward and Parsons (1984*a*) produced a human melanoma cell line that was resistant to MTIC after a single high dose exposure. They found that the resistant line was able to remove $O^6$ methylguanine lesions from its DNA or prevent their formation, much more rapidly than the parent sensitive line. Gibson *et al.* (1986) reported the effects of MTIC on HT29, IMR90, VA13 and BE cell lines. The former two are $Mer^+$, the latter two $Mer^-$. They found equal amounts of DNA strand breaks in the $Mer^+$ and $Mer^-$ lines at equimolar doses, but MTIC was much more toxic to $Mer^-$ lines.

Although strand breaks appear linearly related to MTIC dose, the strand unwinding assay we used and the alkaline elution reported by Gibson *et al.* (1986) did not detect breaks at $25\,\mu mol\,l^{-1}$ MTIC, a concentration producing at least 50% cell kill in $Mer^-$ cell lines. Thus, the failure of 3AAB to potentiate MTIC toxicity in the $Mer^-$ cell line implies that strand breaks or hypersensitivity to strand breaks is not a major mechanism of cell killing at low MTIC concentrations. Hayward and Parsons (1984*b*) also found that an MTIC sensitive melanoma cell line was potentiated far less by another ADPRT inhibitor, 3-aminobenzamide, than the parent resistant line. The use of 3AAB can therefore indicate the relative contributions of different types of DNA damage to cell killing at different MTIC concentrations.

Although the results above suggest the $O^6$MeG lesion in DNA produced by MTIC is a killing lesion, Karran and Williams (1985) found that depleting cells of $O^6$AT with the free methylated base $O^6$MeG did not potentiate the cytotoxicity of nitrosoureas. The lack of potentiation of $Mer^-$ cell lines with 3AAB at low MTIC doses suggests that there may be yet another type of lethal lesion in $Mer^-$ cell lines at low drug concentrations besides $O^6$MeG and strand breaks in DNA, or that very low residual amounts of $O^6$AT are effective. Tisdale, using a novel triazene precursor, found a delayed elevation of ADPRT activity at 2 days in a cell line capable of differentiation. This was probably related to cell cycle effects rather than immediate DNA damage (Tisdale, 1985). The observation that $O^6$MeG is produced in cellular DNA by MTIC and that MTIC can be potentiated by 3AAB provides the basis for *in vivo* studies to potentiate crosslinking nitrosoureas by competing for $O^6$MeG repair, and to potentiate MTIC in $Mer^+$ cells with DNA repair inhibitors. The recent development of precursors to MTIC that are more easily activated than DTIC (Stevens *et al.*, 1984; Wilman *et al.*, 1984; Rutty *et al.*, 1984) will enable this approach to be assessed clinically.

We thank Professor B.T. Golding for synthesis of MTIC. This work was supported by the North of England Cancer Research Campaign.

### References

CARSON, D.A., SETO, S., WASSON, D.B. & CARRERA, C.J. (1986). DNA strand breaks, NAD metabolism and programmed cell death. *Exp. Cell Res.*, **164**, 273.

CAVANAUGH, P.F., JR., PAVELIC, Z.P. & PORTER, C.W. (1984). Enhancement of 1,3-bis (2-chloroethyl)-1-nitrosourea-induced cytotoxicity and DNA damage by α-difluoromethylornithine in L1210 leukemia cells. *Cancer Res.*, **44**, 3856.

CLEAVER, J.E. & MORGAN, W.F. (1985). Poly(ADP-ribose) synthesis is involved in the toxic effects of alkylating agents but does not regulate DNA repair. *Mutat. Res.*, **150**, 69.

COMIS, R.L. (1976). DTIC in malignant melanoma: A perspective. *Cancer Treat. Rep.*, **60**, 165.

CREISSEN, D. & SHALL, S. (1982). Regulation of DNA ligase activity by poly(ADP-ribose). *Nature*, **296**, 271.

DAY, R.S., ZIOLKOWSKI, C.H.J., SCUDIERO, D.A. & 5 others (1980). Defective repair of alkylated DNA by human tumour and SV40-transformed human cell strains. *Nature*, **288**, 724.

DURKACZ, B.W., OMIDIJI, O., GRAY, D.A. & SHALL, S. (1980). (ADP-ribose)$_n$ participates in DNA excision repair. *Nature*, **283**, 593.

ERICKSON, L.C., LAURENT, G., SHARKEY, N.A. & KOHN, K.W. (1980). DNA cross-linking and monoadduct repair in nitrosourea-treated human tumour cells. *Nature*, **288**, 727.

GERSON, S.L., MILLER, K. & BERGER, N.A. (1985). $O^6$ alkylguanine–DNA alkyltransferase activity in human myeloid cells. *J. Clin. Invest.*, **76**, 2106.

GIBSON, N.W., HARTLEY, J., LA FRANCE, R.J. & VAUGHAN, K. (1986). Differential cytotoxicity and DNA-damaging effects produced in human cells of the $Mer^+$ and $Mer^-$ phenotypes by a series of alkyltriazenylimidazoles. *Carcinogenesis*, **7**, 259.

GOTTLIEB, J.A., BENJAMIN, R.S., BAKER, L.H. & 16 others (1976). Role of DTIC (NSC-45388) in the chemotherapy of sarcomas. *Cancer Treat. Rep.*, **60**, 199.

GRAFSTROM, R.C., PEGG, A.E., TRUMP, B.F. & HARRIS, C.C. (1984). $O^6$-alkylguanine-DNA alkyltransferase activity in normal human tissues and cells. *Cancer Res.*, **44**, 2855.

HARRIS, A.L., KARRAN, P. & LINDAHL, T. (1983). $O^6$-methylguanine-DNA methyltransferase of human lymphoid cells: Structural and kinetic properties and absence in repair-deficient cells. *Cancer Res.*, **43**, 3247.

HAYWARD, I.P. & PARSONS, P.G. (1984*a*). Comparison of virus reactivation, DNA base damage, and cell cycle effects in autologous human melanoma cells resistant to methylating agents. *Cancer Res.*, **44**, 55.

HAYWARD, I.P. & PARSONS, P.G. (1984*b*). Epigenetic effects of the methylating agent 5-(3-methyl-1-triazeno) imidazole-4-carboxamide in human melanoma cells. *Aust. J. Exp. Biol. Med. Sci.*, **62**, 597.

KARRAN, P. & WILLIAMS, S.A. (1985). The cytotoxic and mutagenic effects of alkylating agents on human lymphoid cells are caused by different DNA lesions. *Carcinogenesis*, **6**, 789.

MEER, L., JANZER, R.C., KLEIHUES, P. & KOLAR, G.J. (1986). *In vivo* metabolism and reaction with DNA of the cytostatic agent 5-(3,3-dimethyl-1-triazeno)imidazole-4-carboxamide (DTIC). *Biochem. Pharmacol.*, **35**, 3243.

MORAN, M.F. & EBISUZAKI, K. (1985). Inhibition of poly(ADP-ribose) polymerase causes increased DNA strand breaks without decreasing strand rejoining in alkylated HeLa cells. *FEBS Lett.*, **190**, 279.

MYRNES, B., EGGSET, G., VOLDEN, G. & KROKAN, H. (1984*a*). Enzymatic repair of premutagenic DNA lesions in human epidermis. Quantitation of $O^6$-methylguanine–DNA methyltransferase and uracil-DNA glycosylase activities. *Mutat. Res.*, **131**, 183.

MYRNES, B. NORSTRAND, K., GIERCKSKY, K.-E., SJUNNESKOG, C. & KROKAN, H. (1984*b*). A simplified assay for $O^6$-methylguanine-DNA methyltransferase activity and its application to human neoplastic and non-neoplastic tissues. *Carcinogenesis*, **5**, 1061.

NISSELBAUM, J.S. & GREEN, S. (1969). A simple ultramicro method for determination of pyridine nucleotides in tissues. *Anal. Biochem.*, **27**, 212.

OLSSON, M. & LINDAHL, T. (1980). Repair of alkylated DNA in *E. coli*: methyl group transfer from $O^6$-methylguanine to a protein cysteine residue. *J. Biol. Chem.*, **225**, 10569.

PARSONS, P.G., SMELLIE, S.G. MORRISON, L.E. & HAYWARD, I.P. (1982). Properties of human melanoma cells resistant to 5-(3′,3′-dimethyl-1-triazeno)imidazole-4-carboxamide and other methylating agents. *Cancer Res.*, **42**, 1454.

PREUSSMAN, R. & VON HODENBERG, A. (1970). Mechanism of carcinogenesis with 1-aryl-3,3-dialkyltriazenes – II. *In vitro* alkylation of guanosine, RNA and DNA with aryl-monoalkyltriazenes to form 7-alkylguanine. *Biochem. Pharmacol.*, **19**, 1505.

PURNELL, M.R. & WHISH, W.J.D. (1980). Novel inhibitors of poly(ADP-ribose)synthetase. *Biochem. J.*, **185**, 775.

RUTTY, C.J., VINCENT, R.B., ABEL, G., GODDARD, P.M. & HARRAP, K.R. (1984). Studies on the pharmacokinetics and metabolism of some dimethylphenyltriazenes. *Br. J. Cancer*, **50**, 265 (*abstract*).

SCUDIERO, D.A., MEYER, S.A., CLATTERBUCK, B.E., MATTERN, M.R., ZIOLKOWSKI, C.H.J. & DAY, R.S., III (1984). Sensitivity of human cell strains having different abilities to repair $O^6$-methylguanine in DNA to inactivation by alkylating agents including chloroethylnitrosoureas. *Cancer Res.*, **44**, 2467.

SHALL, S. (1982). ADP-ribose in DNA repair. In *ADP-Rybosylation Reactions: Biology and Medicine*, Hayaishi, O. & Ueda, K. (eds) p. 477. Academic Press: London.

SIMS, J.L., BERGER, S.J. & BERGER, N.A. (1983). Poly(ADP-ribose) polymerase inhibitors preserve nicotinamide adenine dinucleotide and adenosine 5′-triphosphate pools in DNA-damaged cells: Mechanism of stimulation of unscheduled DNA synthesis. *Biochemistry*, **22**, 5188.

SKIDMORE, C.J., DAVIES, M.I. GOODWIN, P.M. & 4 others (1979). The involvement of poly(ADP-ribose) polymerase in the degradation of NAD caused by $\gamma$-radiation and N-methyl-N-nitrosourea. *Eur. J. Biochem.*, **101**, 135.

STEVENS, M.F.G., HICKMAN, J.A., STONE, R. & 4 others (1984). Antitumour imidazotetrazines. I. Synthesis and chemistry of 8-carbamoyl-3-(2-chloroethyl) imidazo [5, 1-d]-1, 2, 3, 5-tetrazin-4(3H)-one, a novel broad spectrum antitumour agent. *J. Med. Chem.*, **27**, 196.

TISDALE, M.J. (1985). Antitumour imidazotetrazines – XI. Effect of 8-carbamoyl-3-methylimidazo [5, 1-d]-1, 2, 3, 5-tetrazin-4 (3H)-one [CCRG 81045; M and B 39831 NSC 362856] on poly(ADP-ribose) metabolism. *Br. J. Cancer*, **52**, 789.

UMBENHAUER, D., WILD, C.P., MONTESANO, R. & 7 others (1985). $O^6$-methyldeoxyguanosine in oesophageal DNA among individuals at high risk of oesophageal cancer. *Int. J. Cancer*, **36**, 661.

WALDSTEIN, E.A., CAO, E.H., MILLER, M.E. CRONKITE, E.P. & SETLOW, R.B. (1982). Extracts of chronic lymphocytic leukemia lymphocytes have a high level of DNA repair activity for $O^6$-methylguanine. *Proc. Natl Acad. Sci. USA*, **79**, 4786.

WALKER, I.G., TH'NG, J.P.H., SCHRADER, T.J. & NORRY, T.W. (1984). 3-Aminobenzamide does not increase repair patch size in mammalian cells. *Can. J. Biochem. Cell Biol.*, **62**, 329.

WANI, A.A., WANI, G. & D'AMBROSIO, S.M. (1985). Repair of DNA O-alkylation damage by various huamn organs. *Biochem. Biophys. Res. Commun.*, **133**, 589.

WIESTLER, O., KLEIHUES, P. & PEGG, A.E. (1984). $O^6$-alkylguanine-DNA alkyltransferase activity in human brain and brain tumours. *Carcinogenesis*, **5**, 121.

WILMAN, D.E.V., COX, P.J., GODDARD, P.M., HART, L.I., MERA, K. & NEWELL, D.R. (1984). Tumour inhibitory triazenes. 3. Dealkylation within an homologous series and its relation to antitumour activity. *J. Med. Chem.*, **27**, 870.

WINTERSBERGER, U. & WINTERSBERGER, E. (1985). Poly ADP-ribosylation – A cellular emergency reaction? *FEBS Lett.*, **188**, 189.


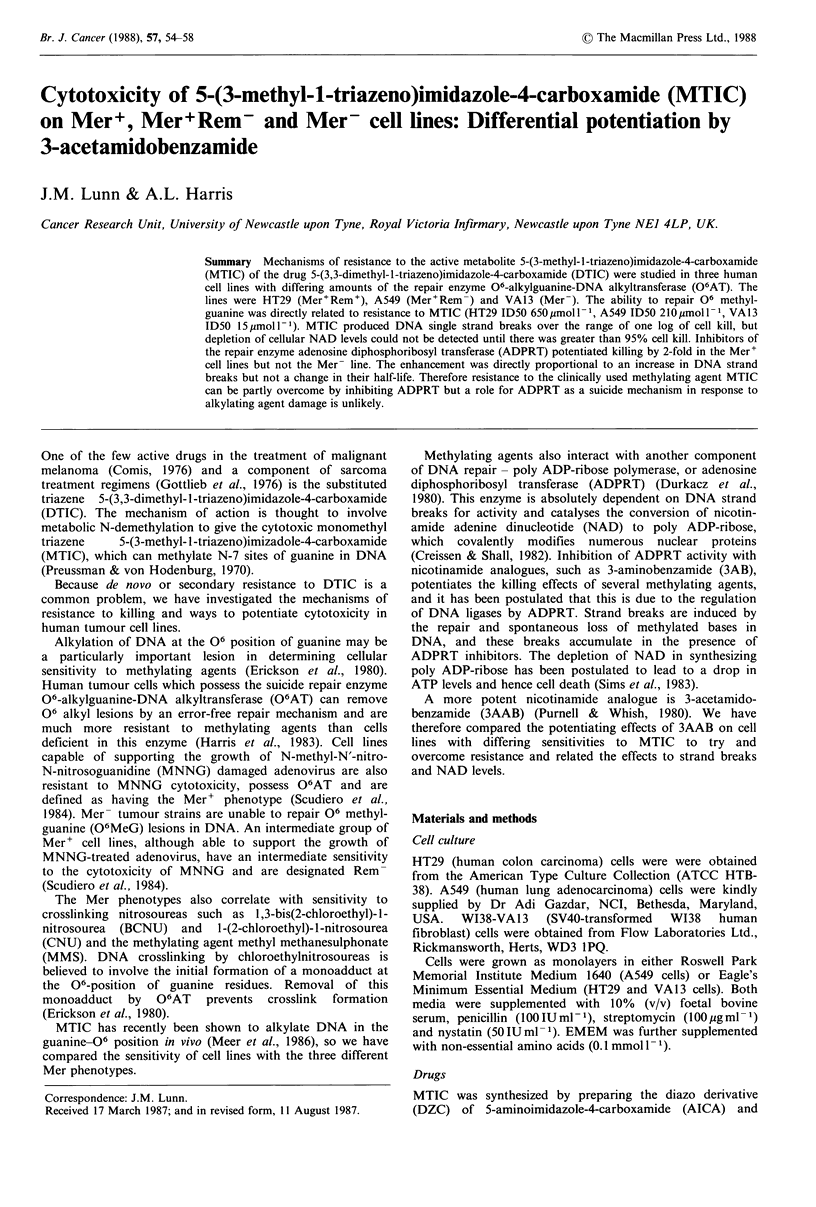

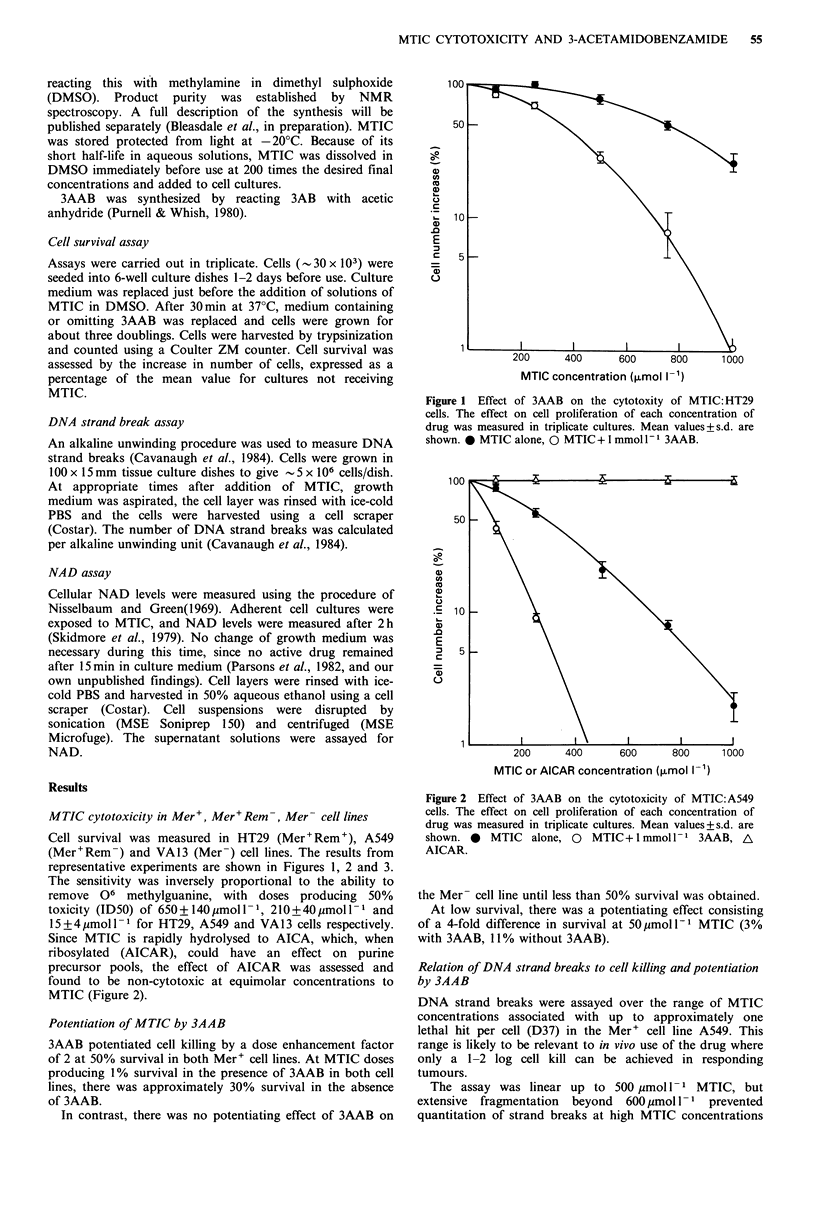

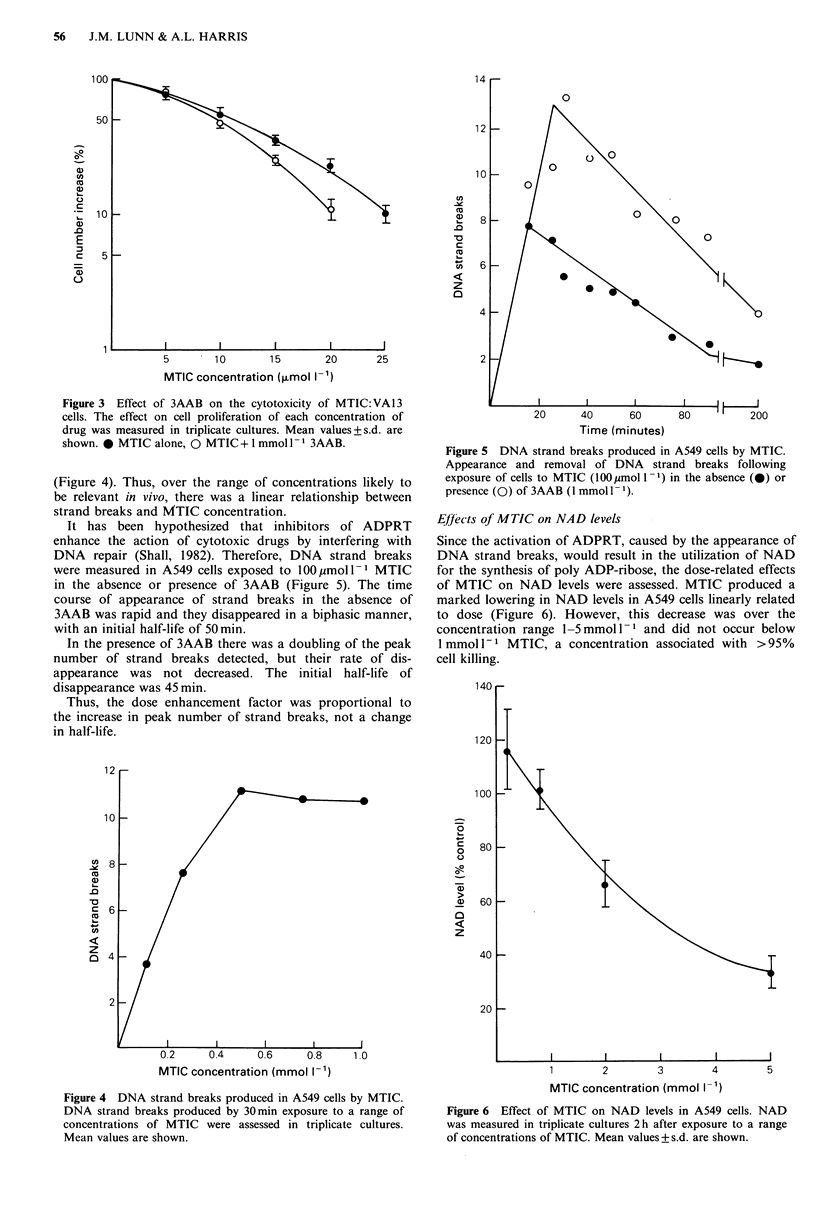

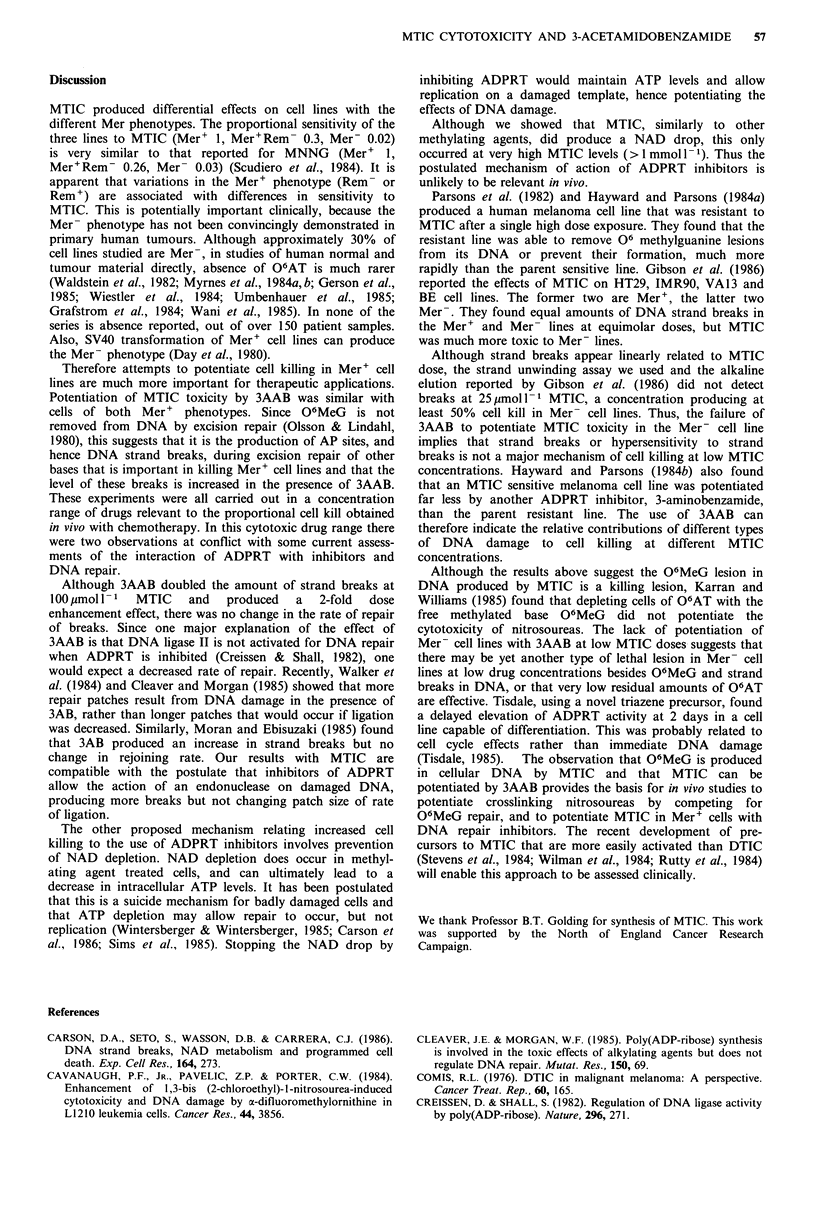

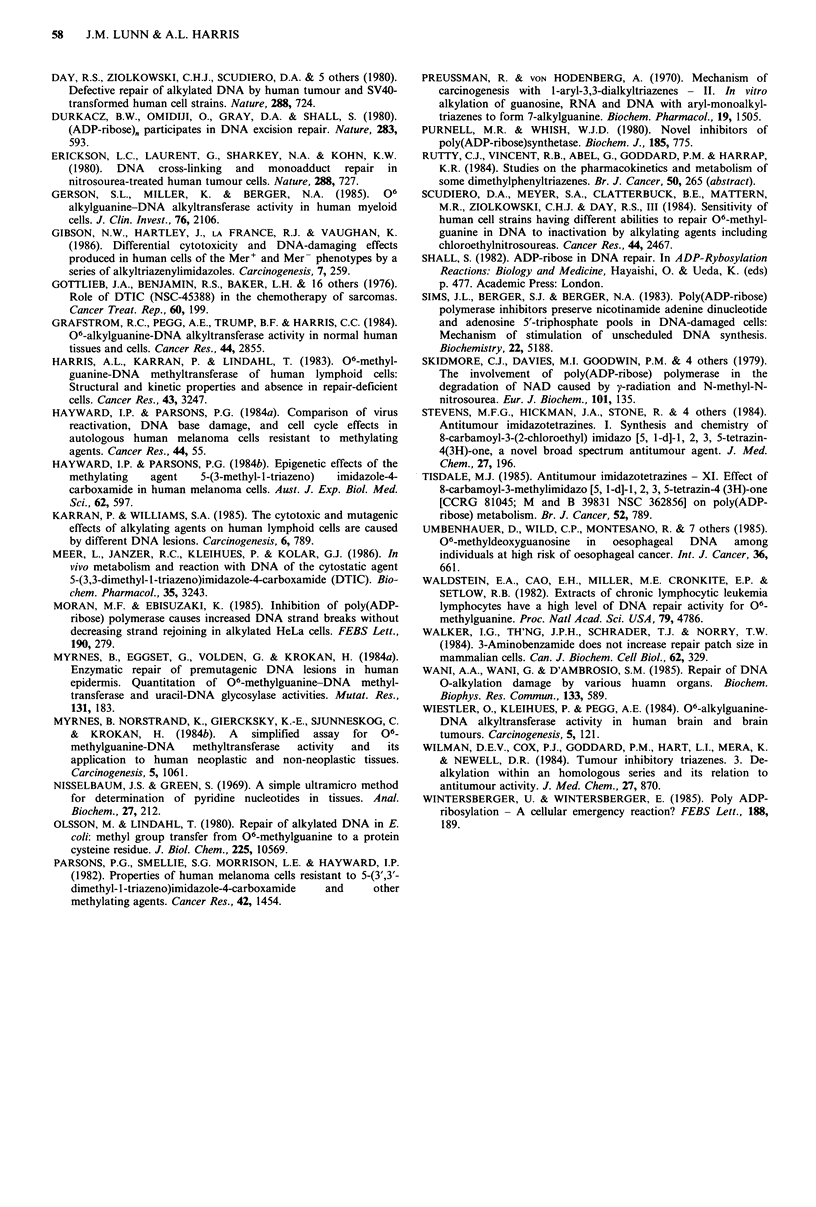


## References

[OCR_00564] Carson D. A., Seto S., Wasson D. B., Carrera C. J. (1986). DNA strand breaks, NAD metabolism, and programmed cell death.. Exp Cell Res.

[OCR_00569] Cavanaugh P. F., Pavelic Z. P., Porter C. W. (1984). Enhancement of 1,3-bis(2-chloroethyl)-1-nitrosourea-induced cytotoxicity and DNA damage by alpha-difluoromethylornithine in L1210 leukemia cells.. Cancer Res.

[OCR_00575] Cleaver J. E., Morgan W. F. (1985). Poly(ADP-ribose) synthesis is involved in the toxic effects of alkylating agents but does not regulate DNA repair.. Mutat Res.

[OCR_00580] Comis R. L. (1976). DTIC (NSC-45388) in malignant melanoma: a perspective.. Cancer Treat Rep.

[OCR_00584] Creissen D., Shall S. (1982). Regulation of DNA ligase activity by poly(ADP-ribose).. Nature.

[OCR_00590] Day R. S., Ziolkowski C. H., Scudiero D. A., Meyer S. A., Lubiniecki A. S., Girardi A. J., Galloway S. M., Bynum G. D. (1980). Defective repair of alkylated DNA by human tumour and SV40-transformed human cell strains.. Nature.

[OCR_00595] Durkacz B. W., Omidiji O., Gray D. A., Shall S. (1980). (ADP-ribose)n participates in DNA excision repair.. Nature.

[OCR_00600] Erickson L. C., Laurent G., Sharkey N. A., Kohn K. W. (1980). DNA cross-linking and monoadduct repair in nitrosourea-treated human tumour cells.. Nature.

[OCR_00605] Gerson S. L., Miller K., Berger N. A. (1985). O6 alkylguanine-DNA alkyltransferase activity in human myeloid cells.. J Clin Invest.

[OCR_00610] Gibson N. W., Hartley J., La France R. J., Vaughan K. (1986). Differential cytotoxicity and DNA-damaging effects produced in human cells of the Mer+ and Mer- phenotypes by a series of alkyltriazenylimidazoles.. Carcinogenesis.

[OCR_00616] Gottlieb J. A., Benjamin R. S., Baker L. H., O'Bryan R. M., Sinkovics J. G., Hoogstraten B., Quagliana J. M., Rivkin S. E., Bodey G. P., Rodriguez V. (1976). Role of DTIC (NSC-45388) in the chemotherapy of sarcomas.. Cancer Treat Rep.

[OCR_00621] Grafstrom R. C., Pegg A. E., Trump B. F., Harris C. C. (1984). O6-alkylguanine-DNA alkyltransferase activity in normal human tissues and cells.. Cancer Res.

[OCR_00626] Harris A. L., Karran P., Lindahl T. (1983). O6-Methylguanine-DNA methyltransferase of human lymphoid cells: structural and kinetic properties and absence in repair-deficient cells.. Cancer Res.

[OCR_00632] Hayward I. P., Parsons P. G. (1984). Comparison of virus reactivation, DNA base damage, and cell cycle effects in autologous human melanoma cells resistant to methylating agents.. Cancer Res.

[OCR_00638] Hayward I. P., Parsons P. G. (1984). Epigenetic effects of the methylating agent 5-(3-methyl-1-triazeno) imidazole-4-carboxamide in human melanoma cells.. Aust J Exp Biol Med Sci.

[OCR_00644] Karran P., Williams S. A. (1985). The cytotoxic and mutagenic effects of alkylating agents on human lymphoid cells are caused by different DNA lesions.. Carcinogenesis.

[OCR_00649] Meer L., Janzer R. C., Kleihues P., Kolar G. F. (1986). In vivo metabolism and reaction with DNA of the cytostatic agent, 5-(3,3-dimethyl-1-triazeno)imidazole-4-carboxamide (DTIC).. Biochem Pharmacol.

[OCR_00655] Moran M. F., Ebisuzaki K. (1985). Inhibition of poly(ADP-ribose)polymerase causes increased DNA strand breaks without decreasing strand rejoining in alkylated HeLa cells.. FEBS Lett.

[OCR_00661] Myrnes B., Eggset G., Volden G., Krokan H. (1984). Enzymatic repair of premutagenic DNA lesions in human epidermis. Quantitation of O6-methylguanine-DNA methyltransferase and uracil-DNA glycosylase activities.. Mutat Res.

[OCR_00668] Myrnes B., Norstrand K., Giercksky K. E., Sjunneskog C., Krokan H. (1984). A simplified assay for O6-methylguanine-DNA methyltransferase activity and its application to human neoplastic and non-neoplastic tissues.. Carcinogenesis.

[OCR_00675] Nisselbaum J. S., Green S. (1969). A simple ultramicro method for determination of pyridine nucleotides in tissues.. Anal Biochem.

[OCR_00680] Olsson M., Lindahl T. (1980). Repair of alkylated DNA in Escherichia coli. Methyl group transfer from O6-methylguanine to a protein cysteine residue.. J Biol Chem.

[OCR_00685] Parsons P. G., Smellie S. G., Morrison L. E., Hayward I. P. (1982). Properties of human melanoma cells resistant to 5-(3',3'-dimethyl-1-triazeno)imidazole-4-carboxamide and other methylating agents.. Cancer Res.

[OCR_00691] Preussmann R., von Hodenberg A. (1970). Mechanism of carcinogenesis with 1-aryl-3,3-dialkyltriazenes. II. In vitro-alkylation of guanosine, RNA and DNA with aryl-monoalkyltriazenes to form 7-alkylguanine.. Biochem Pharmacol.

[OCR_00697] Purnell M. R., Whish W. J. (1980). Novel inhibitors of poly(ADP-ribose) synthetase.. Biochem J.

[OCR_00706] Scudiero D. A., Meyer S. A., Clatterbuck B. E., Mattern M. R., Ziolkowski C. H., Day R. S. (1984). Sensitivity of human cell strains having different abilities to repair O6-methylguanine in DNA to inactivation by alkylating agents including chloroethylnitrosoureas.. Cancer Res.

[OCR_00718] Sims J. L., Berger S. J., Berger N. A. (1983). Poly(ADP-ribose) Polymerase inhibitors preserve nicotinamide adenine dinucleotide and adenosine 5'-triphosphate pools in DNA-damaged cells: mechanism of stimulation of unscheduled DNA synthesis.. Biochemistry.

[OCR_00725] Skidmore C. J., Davies M. I., Goodwin P. M., Halldorsson H., Lewis P. J., Shall S., Zia'ee A. A. (1979). The involvement of poly(ADP-ribose) polymerase in the degradation of NAD caused by gamma-radiation and N-methyl-N-nitrosourea.. Eur J Biochem.

[OCR_00731] Stevens M. F., Hickman J. A., Stone R., Gibson N. W., Baig G. U., Lunt E., Newton C. G. (1984). Antitumor imidazotetrazines. 1. Synthesis and chemistry of 8-carbamoyl-3-(2-chloroethyl)imidazo[5,1-d]-1,2,3,5-tetrazin-4(3 H)-one , a novel broad-spectrum antitumor agent.. J Med Chem.

[OCR_00738] Tisdale M. J. (1985). Antitumour imidazotetrazines--XI: Effect of 8-carbamoyl-3-methylimidazo[5,1-d]-1,2,3,5-tetrazin-4(3H)-one [CCRG 81045; M and B 39831 NSC 362856] on poly(ADP-ribose) metabolism.. Br J Cancer.

[OCR_00744] Umbenhauer D., Wild C. P., Montesano R., Saffhill R., Boyle J. M., Huh N., Kirstein U., Thomale J., Rajewsky M. F., Lu S. H. (1985). O(6)-methyldeoxyguanosine in oesophageal DNA among individuals at high risk of oesophageal cancer.. Int J Cancer.

[OCR_00752] Waldstein E. A., Cao E. H., Miller M. E., Cronkite E. P., Setlow R. B. (1982). Extracts of chronic lymphocytic leukemia lymphocytes have a high level of DNA repair activity fo O6-methylguanine.. Proc Natl Acad Sci U S A.

[OCR_00756] Walker I. G., Th'ng J. P., Schrader T. J., Norry T. W. (1984). 3-Aminobenzamide does not increase repair patch size in mammalian cells.. Can J Biochem Cell Biol.

[OCR_00761] Wani A. A., Wani G., D'Ambrosio S. M. (1985). Repair of DNA O-alkylation damage by various human organs.. Biochem Biophys Res Commun.

[OCR_00766] Wiestler O., Kleihues P., Pegg A. E. (1984). O6-alkylguanine-DNA alkyltransferase activity in human brain and brain tumors.. Carcinogenesis.

[OCR_00771] Wilman D. E., Cox P. J., Goddard P. M., Hart L. I., Merai K., Newell D. R. (1984). Tumor inhibitory triazenes. 3. Dealkylation within an homologous series and its relation to antitumor activity.. J Med Chem.

[OCR_00777] Wintersberger U., Wintersberger E. (1985). Poly ADP-ribosylation--a cellular emergency reaction?. FEBS Lett.

